# Study protocol: Cost-effectiveness of transmural nutritional support in malnourished elderly patients in comparison with usual care

**DOI:** 10.1186/1475-2891-9-6

**Published:** 2010-02-10

**Authors:** Floor Neelemaat, Abel Thijs, Jaap C Seidell, Judith E Bosmans, Marian AE van Bokhorst-de van der Schueren

**Affiliations:** 1 Departments of Nutrition and Dietetics, Internal Medicine and EMGO+ institute for Health and Care Research, VU University Medical Center, Amsterdam, The Netherlands; 2 Department of Internal Medicine, VU University Medical Center, Amsterdam, The Netherlands; 3 Department of Nutrition and Health, Institute for Health Sciences, VU University, Amsterdam, The Netherlands; 4 Section of Health Economics & Health Technology Assessment, Department of Health Sciences and EMGO+ Institute for Health and Research, Faculty of Earth and Life Sciences, VU University, Amsterdam, The Netherlands

## Abstract

**Background:**

Malnutrition is a common consequence of disease in older patients. Both in hospital setting and in community setting oral nutritional support has proven to be effective. However, cost-effectiveness studies are scarce. Therefore, the aim of our study is to investigate the effectiveness and cost-effectiveness of transmural nutritional support in malnourished elderly patients, starting at hospital admission until three months after discharge.

**Methods:**

This study is a randomized controlled trial. Patients are included at hospital admission and followed until three months after discharge. Patients are eligible to be included when they are ≥ 60 years old and malnourished according to the following objective standards: Body Mass Index (BMI in kg/m^2^) < 20 and/or ≥ 5% unintentional weight loss in the previous month and/or ≥ 10% unintentional weight loss in the previous six months. We will compare usual nutritional care with transmural nutritional support (energy and protein enriched diet, two additional servings of an oral nutritional supplement, vitamin D and calcium supplementation, and consultations by a dietitian). Each study arm will consist of 100 patients. The primary outcome parameters will be changes in activities of daily living (determined as functional limitations and physical activity) between intervention and control group. Secondary outcomes will be changes in body weight, body composition, quality of life, and muscle strength. An economic evaluation from a societal perspective will be conducted alongside the randomised trial to evaluate the cost-effectiveness of the intervention in comparison with usual care.

**Conclusion:**

In this randomized controlled trial we will evaluate the effect of transmural nutritional support in malnourished elderly patients after hospital discharge, compared to usual care. Primary endpoints of the study are changes in activities of daily living, body weight, body composition, quality of life, and muscle strength. An economic evaluation will be performed to evaluate the cost-effectiveness of the intervention in comparison with usual care.

**Trial registration:**

Netherlands Trial Register (ISRCTN29617677, registered 14-Sep-2005)

## Background

The primary cause of malnutrition in developed countries is disease. Malnutrition is estimated to occur in 25-61% of all elderly patients suffering from a variety of diseases [1,2]. Unintentional weight loss of ≥ 5% in the previous month and/or unintentional weight loss of ≥ 10% in the previous six months and/or a BMI <20 kg/m^2 ^are often used as parameters to identify malnutrition.

Disease related malnutrition is associated with adverse effects on clinical outcome, as has been shown in a large number of studies. These adverse effects vary from impaired wound healing and postoperative complications to mortality [3]. Poor nutritional status has not only been associated with in-hospital adverse effects, but also with adverse effects both pre-admission and post-discharge. These effects include a trend for increased need for re-hospitalization, significantly higher total mortality, a higher general practitioner consultation rate, higher medication prescription rate, longer rehabilitation, an increased need for nursing home admission, increased likelihood of requiring home health care after discharge and early institutionalization [4,5].

So far, randomized clinical trials have shown that additional Oral Nutritional Support (ONS) can be effective in malnourished elderly people, both in the clinical setting and in the community [6]. In hospitalized patients ONS has been shown to reduce weight loss, to shorten hospital stay and to improve functional status in malnourished hospitalized patients. In the community ONS has been shown to increase activities of daily living, to reduce the number of falls and to reduce health care utilization [5,7-10].

Furthermore, a meta-analysis, including 31 studies and almost 2500 patients, showed that protein and energy supplementation led to small changes in weight and, more importantly to reduced mortality (RR 0,67; CI 0,52-0,87). Also, length of hospital stay was reduced by on average 3.3 days (CI -9.64-3.05) [7].

Because nowadays patients spend only a minority of time in hospital and recover at home, it is not very likely that patients' nutritional status will improve during the short period of admission. Therefore, the problem of disease related malnutrition is more and more becoming a post-discharge problem (in this manuscript further referred to as a transmural problem).

For in-hospital patients, studies on cost-effectiveness of nutritional interventions are scarce. In a retrospective cost-analysis of nine randomized controlled trials on nutritional support, the cost savings aggregated between €503 and €11696 per patient in surgical, orthopedic, elderly and stroke patients [6]. A recent observational cohort study showed a cost reduction in patients supplied with ONS of €723 per patient [8]. In a prospective study, a reduction of length of hospital stay with one day was achieved with an investment of €34 per malnourished patient [11].

Cost-effectiveness studies of ONS in the community are lacking and are eagerly awaited for. We do expect that a nutritional intervention in the transmural setting will be accompanied by higher health care costs than usual care, but that these higher costs are negligible compared with the cost-savings they can potentially generate.

The aim of this study is to investigate the cost-effectiveness of transmural nutritional support in malnourished elderly patients after hospital discharge as compared to usual care on changes in activities of daily living. Secondary outcomes include changes in body weight, body composition, quality of life, and muscle strength between intervention and control group.

## Methods

### Design

This study is designed as a randomized controlled trial comparing transmural nutritional support with usual nutritional care. The study design is in accordance with the Declaration of Helsinki and has been approved by the Medical Ethics Committee (METC) of VU University Medical Center.

Patients are eligible for this study when they are ≥ 60 years old and malnourished according to the following objective standards: Body Mass Index (BMI in kg/m^2^) < 20 and/or, ≥ 5% unintentional weight loss in the previous month and/or ≥ 10% unintentional weight loss in the previous six months. We will compare usual nutritional care with transmural nutritional support (energy and protein enriched diet, two additional servings of an oral nutritional supplement, vitamin D and calcium supplementation, and consultations by a dietitian). The primary outcome parameters will be changes in activities of daily living (functional limitations and physical activity) between the intervention and control group. Secondary outcomes will be changes in body weight, body composition, quality of life, and muscle strength. An economic evaluation from a societal perspective will be conducted alongside the randomised trial to evaluate the cost-effectiveness of the intervention versus usual care.

### Feasibility of recruitment and sample size

Earlier studies have shown that 30% of the elderly hospital population is malnourished at admission [12-16]. For a clinically relevant difference of 20% in nutritional and functional status with a statistical significance level of 0.05 and a power of 80%, two groups of 80 patients were calculated to be sufficient.

A pilot study showed that inclusion of 140 malnourished patients per year is feasible. Taking into account an expected refusal rate of 30% at inclusion and loss to follow-up of 10% during the three months following discharge, we aim to include two groups of 100, to be reached in approximately two years.

### Randomisation

A computerized random number generator will be used to assign patients either to the intervention group or the control group. Patients will be randomized in blocks of ten. At the end of the baseline interview and measurements, the primary investigator opens a consecutively numbered opaque envelope containing the patients' group assignment. Participants, research assistant and researcher are no longer blinded for the intervention from this point. Before starting the analyses the researcher (FN) will be re-blinded for patients' group assignment.

### Population, inclusion and exclusion criteria

All elderly patients (≥ 60 years of age, expected length of hospital admission >2 days) newly admitted to the wards of internal medicine, traumatology and vascular surgery of the VU University Medical Center will be screened at admission by a dietitian and/or research assistants of nutritional status. These departments represent the (sub)specialties general internal medicine, rheumatology, gastroenterology, dermatology, nephrology, orthopedics, traumatology and vascular surgery.

Patients will be excluded from the study when they suffer from senile dementia, can not understand the Dutch language or are not able to or willing to give informed consent.

### Nutritional status

Patients are be eligible for this study if they are identified malnourished according to the following criteria:

• Body Mass Index (BMI in kg/m^2^) < 20 and/or

• ≥ 5% unintentional weight loss in the previous month and/or

• ≥ 10% unintentional weight loss in the previous six months.

Weight (in kg to the nearest decimal) is measured (with patients wearing light indoor clothes and no shoes) on a calibrated chair scale (Prior MD-1512), with an accuracy of 0.1 kilogram, at admission and three months after discharge. A correction factor for clothes will be made by deducting weight with 2.0 kilograms for men and 1.3 kilograms for women [17].

BMI is calculated as actual weight in kilograms divided by the square of height in meters.

As measurement of height is often not feasible in this ill, old and frail population, data on height will be retrieved from self-reported height, with an accuracy of 1.0 centimeter. These data will be validated against height derived from knee height measurements (Seca 207, in cm to the nearest decimal) in approximately 800 elderly patients from the same departments at our institute.

### Intervention

Control patients will receive 'usual' nutritional care, i.e. hospital intervention only on referral by the treating physician and without standardized transmural nutritional support.

Patients assigned to the intervention group strategy will receive standardized transmural nutritional support (**Appendix 1**) starting in hospital and to be continued until three months after discharge.

### Procedure

After obtaining patients' informed consent an inventory will be made of nutritional status, nutritional risk profile and possible confounders. This includes the following baseline characteristics:

- sociodemographic data (age, gender, education level, partner status)

- medical history and medical diagnosis

- anthropometry (weight, height, BMI, percentage involuntary weight loss)

- biochemical parameters (CRP, IGF-1, 25(OH)D)

- mental state (MMSE) [18]

- expected care complexity (COMPRI) [19]

Information on disease, disease severity, disease course, treatment and complications will be retrieved from medical records.

Post-discharge practice will be followed and outcome parameters will be collected for all patients at three months after discharge.

### Outcome parameters

Outcome parameters will be measured after inclusion and at three months after discharge.

Primary outcome is change in activities of daily living, determined as functional limitations and physical activities. All outcome parameters that will be measured are listed below.

#### Activities of daily living

Activities of daily living (ADL) will be assessed with a validated questionnaire that measures the degree of difficulties patients experience with six activities: climbing stairs, walking 5 minutes outdoors without resting, getting up and sitting down in a chair, dressing and undressing oneself, using own or public transportation, and cutting one's own toenails [20]. Functional limitations will be assessed using five difficulty categories, ranging from "No I can not' to 'Yes without difficulty'. Total score will be calculated by summing the scores of all activities, ranging from 0 (does not have any difficulties with the activities) to 6 (has difficulties with all activities).

#### Physical performance

The performance test of physical function includes time measures of walking speed, rising from a chair, putting on and taking off a cardigan, and maintaining balance in a tandem stand [21-23]. To test walking performance a 3 meter walking course is created by a measuring line. Patients are instructed to walk to the other end of the course, to turn 180 degrees, and walk back as quickly as possible. Patients are allowed to use a walking aid if necessary. To test the ability to rise from a chair, patients are asked to fold their arms across their chest and to stand up and sit down five times from a standard hospital chair as quickly as possible. For the cardigan test, patients are asked to put on and take off a cardigan as quickly as possible. To test for balance, patients are asked to stand with one foot placed behind the other in a straight line for at least 10 seconds.

A trained research assistant records the total time needed to complete each test.

Patients who complete the walking test, chair test and cardigan test will be assigned scores between 1 and 4, corresponding to the quartiles of time needed to complete the test, with the fastest time scored as 4. Those who cannot complete the test will be assigned a score of 0. Accordingly the maximum score for these three tests ranges from 0-12 points, after adding up the result of these tree tests.

The balance test will be analyzed separately, yet with the same time classification as described above.

#### Physical activity

Physical activity will be assessed with the validated LASA Physical Activity Questionnaire (LAPAQ) [24]. This face-to-face questionnaire covers the frequency and duration of walking outside, cycling, gardening, sports and household activities during the previous two weeks. Walking and bicycling for transportation purposes are considered as common daily activities in The Netherlands, and not as sports activities. For the analyses, the total time spent on physical activity of the last two weeks is used (in minutes per day).

#### Quality of life

Quality of Life will be measured by the SF-12 and EuroQol (EQ-5D and EQ-VAS).

The SF-12 contains 12 questions from the SF-36 [25]. These questions concern functional status -both physical and social functioning-mental health, pain, vitality and evaluation of persons' state of health. With these dimensions two total scores can be calculated; one physical component score and one mental component score [26].

EuroQol (EQ-5D) is a standardised instrument to monitor health outcome [27]. This instrument contains five short questions on mobility, self-care, daily activities, pain/discomfort and anxiety/depression with three possible response categories (no problems, moderate problems, severe problems). The EQ-5D scores were used to calculate utilities using the Dutch tariff [28]. QALYs were calculated by multiplying the utilities with the amount of time a patient spent in a particular health state. Transitions between health states were linearly interpolated.

Additionally we will administer the EQ VAS. The EQ-VAS, a visual analog scale, generates a self-rating of health-related quality of life. The patient rates his/her health state by drawing a line from the box marked "Your health state today" to the appropriate point on the EQ-VAS on a scale of 0 to 100.

#### Hand grip strength

Hand grip strength (in kg) will be measured with a hydraulic hand dynamometer (Baseline, Fabrication Enterprises Inc., Elmsford, NY, USA). Respondents are asked to perform two maximum force trials with their non-dominant hand. The highest value will be used. Measured data will be compared to reference values by Mathiowetz. Data will be expressed as percentage of reference value [29].

#### Fall-incidents

Patients will be asked to report all fall incidents weekly in a fall diary for a period of three months after discharge. A fall is defined as "an unintentional change in position resulting in coming to rest at a lower level or on the ground" [30].

#### Bio-electrical impedance spectroscopy

Bio-electrical impedance spectroscopy (BIS) will be applied to calculate (changes in) body composition. Measurements will be performed at the non-dominant side of the patient, using a Hydra ECF/ICF Bio Impedance Spectrum Analyzer, model 4200 (Xitron Technologies, San Diego, CA, USA). Shoes, socks and jewellery will be removed and patients will be in supine position. Two current electrodes (tetra-polar electrodes (3 M red Dot AG/AgCl)) will be placed at the dorsal surfaces of the hand and foot on the distal position of the second metacarpal and metatarsal, respectively. Two detector electrodes will be placed at the posterior wrist between the styloid processes of the radius and ulna and at the ankle between the tibial and fibular malleoli. With this technique, two body compartments, fat mass (FM) and fat free mass (FFM), can be determined. Patients with a pacemaker will be excluded from this measurement.

#### Resting energy expenditure

Resting energy expenditure (REE) will be calculated from measurements of oxygen consumption (VO_2_) and carbon dioxide production (VCO_2_) by with a ventilated hood system, in supine position for 30 minutes, with a metabolic monitor (Vmax Encore n29, Viasys Healthcare, Houten, The Netherlands). Before each measurement, gas analyzers will be calibrated with two reference gas mixtures (a. 16% O_2_, 4% CO_2_, bal. N_2 _and b. 26% O_2_, bal. N_2_. (Viasys)). Patients will be monitored during the measurement to prevent movements or sleeping under the hood.

REE will be calculated from oxygen consumption and carbon dioxide production by using the equation of Weir [31].

#### Dietary intake

Dietary intake will be obtained by asking in broad outlines for patients' mean daily intake of food and drinks in the two weeks before admission to the hospital and at three months after discharge.

#### Biochemical parameters CRP, IGF-1, 25(OH)D

Two blood samples per patient will be obtained: the first on the day after inclusion and the second three months after discharge. We will measure CRP, IGF-1 and 25(OH)D in both samples. The blood samples will be centrifuged and stored at -80°C. All blood samples will be analyzed simultaneously after completing full data collection.

C-reactive proteïn (CRP in mg/l) is a member of the class of acute-phase reactants as its levels rise dramatically during inflammation processes. Plasma concentrations of C-Reactive Protein (CRP) will be measured with an automated latex-enhanced immunoturbidimetric assay on a Modular P800 analyzer (Roche Diagnostics, Almere, The Netherlands).

Insuline-like growth factor (IGF-1 in μg/l) will be obtained to observe over or under production of growth-hormone. Serum levels of IGF-1 will be measured with a Immulite 2500 analyzer (Siemens, Deerfield, IL, USA)

Serum 25(OH)D (in nmol/l) will be obtained to observe differences in vitamin D levels after the three month supplementation of vitamin D in the intervention group. This will be determined using a competitive protein binding assay (Nichols Diagnostics, San Juan Capistrano, CA, USA).

All analyses will be performed at department of Clinical Chemistry of the VU University Medical Center.

#### Costs

Costs will be measured from a societal perspective. Direct health care costs include the costs of transmural nutritional support (nutritional supplements plus dietitian), hospitalization, additional visits to other health care providers (general practitioner, medical specialist), prescription and over-the-counter medication, professional home care. Direct non-health care costs of paid and unpaid help and indirect cost of absenteeism of paid and unpaid work will also be included.

Data on the use of nutritional support will be registered by the dietician implementing the transmural nutritional intervention. Data on health care utilization during hospitalization will be retrieved from medical records and the hospital information system. Use of other health care resources after discharge from the hospital will be collected through two identical cost diaries each spanning a period of 6 weeks. Medication data will be obtained from the patients' pharmacies.

Health care utilization will be valued using standard costs from the Dutch guidelines for cost analysis in health care research [32]. If there are no standard costs available, tariffs or prices from health care providers themselves or professional organizations will be used. The costs of medication will be estimated on the basis of prices charged by the Royal Dutch Society for Pharmacy.

#### Economic evaluation

The aim of the economic evaluation will be to determine and compare the total costs for patients receiving either transmural nutritional support or usual care, and to relate these costs to the effects of the interventions.

Incremental cost-effectiveness ratios (ICERs) will be calculated by dividing the difference in mean total costs between the two interventions by the difference in mean effects between the two interventions. The ICERs will be calculated for the primary clinical effect measures of the trial, i.e. functional status and ADL. Cost-utility ratios will also be calculated.

Bootstrapping will be used for pair-wise comparison of the mean differences in total costs between the intervention groups. Confidence intervals will be obtained by bias corrected and accelerated (Bca) bootstrapping using 5000 replications [33]. To estimate the uncertainty surrounding the ICERs bias corrected accelerated bootstrapping (5000 replications) will be used. The bootstrapped cost-effect pairs will be plotted on cost-effectiveness planes and will be used to estimate cost-effectiveness acceptability curves.

### Organization

The primary investigator is responsible for the informed consent procedure, final patient selection, measurements, analysis and reports. The primary investigator will be assisted by a research assistant.

Data flow will be controlled by the primary investigator, using an administrative database system. Data-entry and control will be conducted by a research assistant under supervision of the investigator. The primary investigator is responsible for the data cleaning and analysis.

### Statistical analyses

All analyses will be performed according to the intention-to-treat principle. 95% confidence intervals will be calculated for the differences in percentages and means. Logistic regression will be used to analyze dichotomous variables, Poisson regression for the count-variables and linear regression for continuous variables. Ceiling and floor effects will be taken into account in the analysis of the questionnaires.

In order to test the independent contribution of the intervention on the outcome variables, multivariate regression analysis will be used to adjust for the possible confounders.

## Discussion

When designing the study protocol we had to make a number of considerations, which may lead to the following strengths and weaknesses:

Firstly, we have chosen a strict definition of malnutrition, because the most malnourished group is most likely to benefit from oral nutritional support. Since we have used the described cut-off points for malnutrition in previous studies as well, we expect that it is feasible to include a sufficient number of patients in the proposed study.

Secondly, we have chosen not to use strict exclusion criteria, but to include all eligible patients, even though they are suffering from a variety of (chronic) diseases. Their homogeneity stems from their age (>60y), degree of malnutrition (severe malnutrition) and background of disease (mainly non-surgical). We have performed earlier studies with this same group of relatively unselected patients with positive results of the nutritional intervention (after correcting for possible confounders such as age, sex, ethnicity, care complexity, disease etc) in comparison with usual care [11,34]. Moreover, if the results of a broad study like this one are positive, it justifies wide implementation, because the included group is representative for a mixed elderly hospital population; in contrast, selection of a more specific group would make the intervention less applicable to other patients groups.

Thirdly, for research purposes, standardized oral nutritional supplementation is chosen because simply advising patients to increase their intake by eating more has not proven to be a solution that can be relied on. From earlier studies, it is known that providing extra supplements does increase energy intake and leads to weight gain, in contrast to dietary advice alone (without extra supplements) [10,35-37].

Fourthly, the follow-up period of three months is chosen because this seems a reasonable recovery period after discharge in this particular patient group. Also, it is known that compliance of patients to take extra supplements diminishes after three months [38,39]. By making regular phone calls to the patients we try to maintain compliance.

Fifthly, care should be taken of in-hospital contamination between treatment groups. The majority of in-hospital patients will not be supplemented additional nutrition, either because they are not malnourished (± 70%) or because they are randomized to the control strategy (50% of the study population, 15% of the total population). Except for the primary investigator and research assistant, doctors and nurses will not be aware of the reason for not-supplementing. Thus we expect to be able to prevent contamination between treatment groups. In addition, hospital admission will last only 10 to 15 days and post-discharge treatment will be approximately three months. We expect that the post-discharge period to account for the majority of the effects. Contamination between treatment groups in not likely to occur after discharge.

Finally, this is the first prospective, randomized controlled trial evaluating whether transmural nutritional support is effective and cost-effectiveness when compared to usual care.

## Conclusion

In this study we will evaluate in a randomized controlled trial whether transmural nutritional support, compared to usual care, is effective and cost-effective, in malnourished elderly patients. We will determine changes in activities of daily living, physical activity, functional limitations, body weight, body composition, quality of life, and muscle strength between the intervention and control group during the period between admission to hospital and three months after discharge.

## Appendix 1

Standardised transmural nutritional support in the intervention group:

- Energy and protein enriched diet (in-hospital period)

- Two additional servings of an oral nutritional supplement, leading to an expected increase in intake of ± 600 kcal/day and 24 g protein/day per day (entire study period)

- 400 IE vitamin D3 and 500 mg calcium (Calci Chew-D3, Sandoz) per day (entire study period)

- Six consultations by telephone by a dietitian in order to give advice and to stimulate compliance to the proposed nutritional intake (every other week after discharge from the hospital)

## Competing interests

The authors declare that they have no competing interests.

## Authors' contributions

FN, AT, JS, JB, and MvB provided support in the design of the study and contributed input into the main ideas of this manuscript. FN drafted the manuscript and all other authors contributed to the further writing of the manuscript and approved it.

## References

[B1] Klipstein-Grobusch K Reilly JJ Potter J Edwards CA Roberts MA Energy intake and expenditure in elderly patients admitted to hospital with acute illness Br J Nutr 1995 73 2 323 334 10.1079/BJN19950033 7718550

[B2] Watson JL The prevalence of malnutrition in patients admitted to care of the elderly wards Proceedings of the Nutrition Society 1999 58 139A 10.1079/PNS19990019

[B3] Flodin L Svensson S Cederholm T Body mass index as a predictor of 1 year mortality in geriatric patients Clin Nutr 2000 19 2 121 125 10.1054/clnu.1999.0091 10867730

[B4] Martyn CN Winter PD Coles SJ Edington J Effect of nutritional status on use of health care recources by patients with chronic disease living in the community Clin Nutr 1998 17 119 123 10.1016/S0261-5614(98)80005-8 10205328

[B5] Payette H Boutier V Coulombe C Gray-Donald K Benefits of nutritional supplementation in free-living, frail, undernourished elderly people: a prospective randomized community trial J Am Diet Assoc 2002 102 8 1088 1095 12171453

[B6] Stratton RJ Green CJ Elia M Disease related malnutrition: an evidence-based approach to treatment 2003 1 Cambridge: CABI Publishing 1 34

[B7] Milne AC Potter J Avenell A Protein and energy supplementation in elderly people at risk from malnutrition Cochrane Database Syst Rev 2002 3 CD003288 12137686 10.1002/14651858.CD003288

[B8] Arnaud-Battandier F Malvy D Jeandel C Schmitt C Aussage P Beaufrere B Cynober L Use of oral supplements in malnourished elderly patients living in the community: a pharmaco-economic study Clin Nutr 2004 23 5 1096 1103 10.1016/j.clnu.2004.02.007 15380901

[B9] Baldwin C Parsons TJ Dietary advice and nutritional supplements in the management of illness-related malnutrition: systematic review Clin Nutr 2004 23 6 1267 1279 10.1016/j.clnu.2004.07.018 15556249

[B10] Baldwin C Weekes CE Dietary advice for illness-related malnutrition in adults Cochrane Database Syst Rev 2008 1 CD002008 18254000 10.1002/14651858.CD002008.pub3

[B11] Kruizenga HM Van Tulder MW Seidell JC Thijs A Van Bokhorst-de van der Schueren MAE Effectiveness and cost-effectiveness of early screening and treatment of malnourished patients Am J Clin Nutr 2005 82 1082 1089 16280442 10.1093/ajcn/82.5.1082

[B12] Kruizenga HM Wierdsma NJ van Bokhorst MA Hollander HJ Jonkers-Schuitema CF Heijden E van der Melis GC van Staveren WA Screening of nutritional status in The Netherlands Clin Nutr 2003 22 2 147 152 10.1054/clnu.2002.0611 12706131

[B13] Klinkenberg M Binsbergen van JJ Strack van Schijndel RJM Thijs A Bokhorst-de van der Schueren MAE Gegevensoverdracht rond ondervoede patiënt: berichtgeving is mager Huisarts en wetenschap 2005 48 5 216 219

[B14] van Bokhorst-de van der Schueren MA Klinkenberg M Thijs A Profile of the malnourished patient Eur J Clin Nutr 2005 59 10 1129 1135 10.1038/sj.ejcn.1602222 16015259

[B15] Kruizenga HM Seidell JC de Vet HC Wierdsma NJ van Bokhorst-de van der Schueren MAE Development and validation of a hospital screening tool for malnutrition: the short nutritional assessment questionnaire (SNAQ) Clin Nutr 2005 24 1 75 82 10.1016/j.clnu.2004.07.015 15681104

[B16] Halfens RJG Janssen MAP Meijers JMM Wansink SW Landelijke Prevalentiemeting Decubitus en andere zorgproblemen: Herziene resultaten zevende jaarlijkse meting 2004 Universiteit Maastricht 2004

[B17] Frank E Dunlop AL What does a patient's outfit weight? Fam Med 2000 32 9 595 596 11039142

[B18] Folstein MF Folstein SE McHugh PR "Mini-mental state". A practical method for grading the cognitive state of patients for the clinician J Psychiatr Res 1975 12 3 189 198 10.1016/0022-3956(75)90026-6 1202204

[B19] de Jonge P Huyse FJ Slaets JPJ Herzog T Lobo A Lyons JS Care complexity in the general hospital. Results from a European study Psychosomatics 2001 42 3 204 212 10.1176/appi.psy.42.3.204 11351108

[B20] Kriegsman DM Deeg DJ van Eijk JT Penninx BW Boeke AJ Do disease specific characteristics add to the explanation of mobility limitations in patients with different chronic diseases? A study in The Netherlands J Epidemiol Community Health 1997 51 6 676 685 10.1136/jech.51.6.676 9519132 PMC1060566

[B21] Penninx BW Deeg DJ van Eijk JT Beekman AT Guralnik JM Changes in depression and physical decline in older adults: a longitudinal perspective J Affect Disord 2000 61 1-2 1 12 10.1016/S0165-0327(00)00152-X 11099735

[B22] Guralnik JM Simonsick EM Ferrucci L Glynn RJ Berkman LF Blazer DG A short physical performance battery assessing lower extremity function: association with self-reported disability and prediction of mortality and nursing home admission J Gerontol 1994 49 2 M85 M94 8126356 10.1093/geronj/49.2.m85

[B23] Guralnik JM Ferrucci L Simonsick EM Salive ME Wallace RB Lower-extremity function in persons over the age of 70 years as a predictor of subsequent disability N Engl J Med 1995 332 9 556 561 10.1056/NEJM199503023320902 7838189 PMC9828188

[B24] Stel VS Smit JH Pluijm SM Visser M Deeg DJ Lips P Comparison of the LASA Physical Activity Questionnaire with a 7-day diary and pedometer J Clin Epidemiol 2004 57 3 252 258 10.1016/j.jclinepi.2003.07.008 15066685

[B25] Ware JE Gandek B Kosinski M Aaronson NK Apolone G Brazier J The equivalence of SF-36 summary health scores estimatedusing standard and country-specific algorithms in 10 countries: results from the IQOLA project J Clin Epidemiol 1998 51 11 1167 1170 10.1016/S0895-4356(98)00108-5 9817134

[B26] Ware JEJr Kosinski M Bayliss MS McHorney CA Rogers WH Raczek A Comparison of methods for the scoring and statistical analysis of SF-36 health profile and summary measures: summary of results from the Medical Outcomes Study Med Care 1995 33 4 Suppl AS264 AS279 7723455

[B27] Szende A Oppe M Devlin N EQ-5D Value Sets: Inventory, Comparative Review and User Guide 1995

[B28] Lamers LM Stalmeier PF McDonnell J Krabbe PF van Busschbach JJ Measuring the quality of life in economic evaluations: the Dutch EQ-5D tariff Ned Tijdschr Geneeskd 2005 149 28 1574 1578 16038162

[B29] Mathiowetz V Kashman N Volland G Weber K Grip and pinch strength: normative data for adults Arch Phys Med Rehabil 1985 66 69 74 3970660

[B30] The prevention of falls in later life A report of the Kellogg International Work Group on the prevention of falls by the elderly Dan Med Bull 1987 34 Suppl 4 1 24 3595217

[B31] Weir JB New methods for calculating metabolic rate with special reference to protein metabolism J Physiol 1949 109 1-2 1 9 15394301 10.1113/jphysiol.1949.sp004363 PMC1392602

[B32] Oostenbrink JB Bouwmans CAM Koopmanschap MA Rutten FFH Guidelines for cost studies, methods and standard costs for economic evaluations in health care Dutch Health Insurance Council 2004

[B33] Efron B Tibshirani RJ An introduction to the bootstrap 1993 New York London: Chapman & Hall

[B34] Kruizenga HM de Jonge P Seidell JC Neelemaat F van Bodegraven AA Wierdsma NJ van Bokhorst-de van der Schueren MAE Are malnourished patients complex patients? Health status and care complexity of malnourished patients detected by the Short Nutritional Assessment Questionnaire (SNAQ) Eur J Intern Med 2006 17 3 189 194 10.1016/j.ejim.2005.11.019 16618452

[B35] Edington J Barnes R Bryan F Dupree E Frost G Hickson M A prospective randomised controlled trial of nutritional supplementation in malnourished elderly in the community: clinical and health economic outcomes Clin Nutr 2004 23 2 195 204 10.1016/S0261-5614(03)00107-9 15030959

[B36] Joosten E Elst EB van der Does nutritional supplementation influence the voluntary dietary intake in an acute geriatric hospitalized population? Aging 2001 13 5 391 394 11820713 10.1007/BF03351508

[B37] Baldwin C Parsons T Logan S Dietary advice for illness-related malnutrition in adults Cochrane Database Syst Rev 2001 2 CD002008 11406021 10.1002/14651858.CD002008

[B38] Gosney M Are we wasting our money on food supplements in elder care wards? J Adv Nurs 2003 43 3 275 280 10.1046/j.1365-2648.2003.02710.x 12859786

[B39] Kayser-Jones J Schell ES Porter C Barbaccia JC Steinbach C Bird WF Redford M Pengilly K A prospective study of the use of liquid oral dietary supplements in nursing homes J Am Geriatr Soc 1998 46 11 1378 1386 9809759 10.1111/j.1532-5415.1998.tb06004.x

